# Biometric covariates and outcome in COVID-19 patients: are we looking close enough?

**DOI:** 10.1186/s12879-021-06823-z

**Published:** 2021-11-04

**Authors:** Konstantin Sharafutdinov, Sebastian Johannes Fritsch, Gernot Marx, Johannes Bickenbach, Andreas Schuppert

**Affiliations:** 1 Institute for Computational Biomedicine, RWTH Aachen University, Pauwelsstr. 19, 52074 Aachen, Germany; 2 Joint Research Center for Computational Biomedicine, RWTH Aachen University, Pauwelsstr. 19, 52074 Aachen, Germany; 3 Department of Intensive Care Medicine, University Hospital RWTH Aachen, Pauwelsstr. 30, 52074 Aachen, Germany; 4 Juelich Supercomputing Centre, Forschungszentrum Juelich, Wilhelm-Johnen-Straße, 52428 Jülich, Germany

**Keywords:** COVID-19, SARS-CoV2, Risk factors, Biometric covariates

## Abstract

**Background:**

The impact of biometric covariates on risk for adverse outcomes of COVID-19 disease was assessed by numerous observational studies on unstratified cohorts, which show great heterogeneity. However, multilevel evaluations to find possible complex, e.g. non-monotonic multi-variate patterns reflecting mutual interference of parameters are missing. We used a more detailed, computational analysis to investigate the influence of biometric differences on mortality and disease evolution among severely ill COVID-19 patients.

**Methods:**

We analyzed a group of COVID-19 patients requiring Intensive care unit (ICU) treatment. For further analysis, the study group was segmented into six subgroups according to Body mass index (BMI) and age. To link the BMI/age derived subgroups with risk factors, we performed an enrichment analysis of diagnostic parameters and comorbidities. To suppress spurious patterns, multiple segmentations were analyzed and integrated into a consensus score for each analysis step.

**Results:**

We analyzed 81 COVID-19 patients, of whom 67 required mechanical ventilation (MV). Mean mortality was 35.8%. We found a complex, non-monotonic interaction between age, BMI and mortality. A subcohort of patients with younger age and intermediate BMI exhibited a strongly reduced mortality risk (p < 0.001), while differences in all other groups were not significant. Univariate impacts of BMI or age on mortality were missing. Comparing MV with non-MV patients, we found an enrichment of baseline CRP, PCT and D-Dimers within the MV group, but not when comparing survivors vs. non-survivors within the MV patient group.

**Conclusions:**

The aim of this study was to get a more detailed insight into the influence of biometric covariates on the outcome of COVID-19 patients with high degree of severity. We found that survival in MV is affected by complex interactions of covariates differing to the reported covariates, which are hidden in generic, non-stratified studies on risk factors. Hence, our study suggests that a detailed, multivariate pattern analysis on larger patient cohorts reflecting the specific disease stages might reveal more specific patterns of risk factors supporting individually adapted treatment strategies.

**Supplementary Information:**

The online version contains supplementary material available at 10.1186/s12879-021-06823-z.

## Background

The novel Severe acute respiratory syndrome Coronavirus 2 (SARS-CoV2)-infection COVID-19 most commonly presents with mild symptoms [1]. Among German patients, hospitalization is necessary only in a small proportion of cases. Those in need of inhouse treatment with COVID-19 can often be handled at general wards while only a minority of patients with a fulminant deterioration is in need for intensive care resources and consecutive ventilatory support [2]. However, this particular patient group predominantly exhibiting Acute respiratory distress syndrome (ARDS) and severe hypoxia requires complex and extensive treatment [3]. For a targeted use of the available intensive care beds, it is of great importance to know which patients are particularly at risk of suffering a severe course. The impact of biometric covariates on risk for severe outcomes of COVID-19 disease was assessed by numerous observational studies on unstratified cohorts [4–7]. Several international publications identified older age, male gender and increased number of comorbidities as risk factors for a poor outcome [8, 9]. However, existing studies differ in both clinical outcomes under consideration and estimated influence of single biometric parameters. Conditions of hospitalization and Intensive care unit (ICU) resources differ [4, 6, 7, 10, 11], which makes a reasonable comparison between the studies difficult. Possible reasons for different outcomes may lay in large heterogeneities of health care systems, hospital and especially ICU resources as well as differing admission policies and clinical operating instructions. Another obstacle for a clear picture is the considerably differing populations under analysis between the studies. Some studies included only hospitalized and deceased patients, while in other publications, non-hospitalized patients with milder courses of disease were included, which led to different estimates for the impact of biometric covariates, as well. However, multilevel evaluations, which are able to find possible complex, e.g. non-monotonic multi-variate patterns reflecting mutual interference of parameters, are usually missing. The fact that there is a lack of clarity about the real significance of certain factors has led to uncertainty and partial rejection of prophylactic protective measures in parts of the public [12]. To elucidate the impact of different biometric risk factors on the course of the novel disease, it might be necessary to go into a more detailed, computational analysis.

To give an example of these more advanced techniques, we performed a retrospective analysis of a dataset of COVID-19 patients. Within this study, we aimed to investigate the influence of biometric differences on mortality and disease evolution within a cohort of severely ill COVID-19 patients.

## Methods

This analysis was approved by the local ethical review board (EK 091/20; Ethics Committee, Faculty of Medicine, RWTH Aachen, Aachen, Germany). The Ethics Committee waived the need to obtain Informed consent for the collection, analysis and publication of the retrospectively obtained and depersonalized data.

The dataset included patients with a confirmed diagnosis of COVID-19 between March and May 2020, who were admitted to the University Hospital RWTH Aachen and needed treatment on an ICU. This analysis was conducted retrospectively on data obtained for clinical purposes during the ICU treatment. Thus, the individual observation ended with discharge from the ICU. An additional follow-up after discharge was not carried out.

Data were retrieved from an electronic patient data recording system (medico//s, Siemens, Germany) and from an online patient data documentary system (IntelliSpace Critical Care and Anesthesia, ICCA Rev. F.01.01.001, Philips Electronics, The Netherlands). All data were merged into a data register (Excel, Version 16.37, Microsoft Corporation, Redmond, WA, USA).

Acute respiratory distress syndrome (ARDS) and its severity was classified according to the grades of hypoxia as defined by the “Berlin definition” [13].

The recorded data contained biometric parameters like age and gender, preexisting comorbidities and a set of 54 diagnostic baseline value parameters assessed in the ICU, i.e. the first available measurements of respective parameters containing vital signs, laboratory parameters and the ventilator settings (see Additional file 1). ICU mortality was chosen as primary endpoint, while length of MV represented a secondary endpoint.

Since age and body mass index (BMI) were described previously as relevant for the evolution of disease, they were particularly chosen for a more detailed analysis. For the analysis of the interactions between BMI, age and mortality, the population was divided into three BMI subgroups, defined as low, intermediate and high BMI group and a lower and a higher age group. In order to minimize spurious effects from subgroup splitting, we analyzed subgroups across a bundle of subgroup settings. The respective splitting value were 25 and 27 for the lower and 30 and 32 for the upper limit. Age splitting was carried out at the bounds 65, 68 and 70 years. These splitting resulted in 12 possible combinations of subgroups, which were analyzed individually. To avoid very small subgroups, no further splitting with respect to gender was carried out. The 12 possible combinations, which resulted from applying variable subgroup borders, were summarized in 6 subcohorts by calculating a mean value for the different groups with a combination from BMI (low/intermediate/high) and age (low/high). By that, outcomes and identification of diagnostic parameters, which are specific for each of the 6 subgroups, were calculated. To identify risk factors associated with the diverse outcome across the biometric subcohorts, we calculated differential expression for comorbidities and diagnostic baseline value parameters. All statistical analyses were performed using Matlab R2015b, Statistical and Machine Learning Toolbox (The MathWorks, Inc.).

Primarily, the impact of BMI and age on mortality was analyzed. Second, the distribution of age and BMI in survivors and non-survivors was further calculated individually (Wilcoxon-Ranksum test). Based on these results, we stratified BMI into three subgroups as described above and analyzed the age distribution of survivors and non-survivors in each subcohort. The same analysis was performed for length of MV as secondary endpoint, followed by statistical analysis of relation between the two endpoints. Finally, analogous calculation was performed comparing a cohort of non-ventilated ICU patients with a ventilated cohort. To complete the analysis, we analyzed the enrichment of comorbidities as well as differential expression of the baseline diagnostic parameters between MV survivors with length of MV below 30 days and more than 30 days, respectively. Additionally, for each subcohort we performed enrichment for mortality using hypergeometric cumulative distribution testing (Matlab: hygecdf).

## Results

### Patient characteristics

We analyzed data of 81 COVID-19 patients, of whom 67 required MV during their ICU stay. Clinical characteristics of the complete population are shown in Table 1.

**Table 1:** Clinical characteristics of the analyzed ICU patient cohort

	Total
Total number of patients, n (%)	81 (100)
Age (mean ± SD), years	64.3 ± 11.3
Male gender, n (%)	54 (66.6)
Length of stay ICU, days (mean ± SD)	25.8 ± 22.0
MV, n (%)	67 (83)
Length of MV, days (mean ± SD)	26.5 ± 20.6
ARDS, n (%)	57 (70.4)
Mild, n (%)	1 (1.2)
Moderate, n (%)	21 (25.9)
Severe, n (%)	35 (43.2)
Pulmonary Embolism, n (%)	17 (21.0)
Renal replacement therapy, n (%)	46 (56.8)
ECMO, n (%)	16 (19.8)
Mortality, n (%)	29 (35.8)

### Influence of BMI and age on mortality and length of MV

**Fig. 1:**
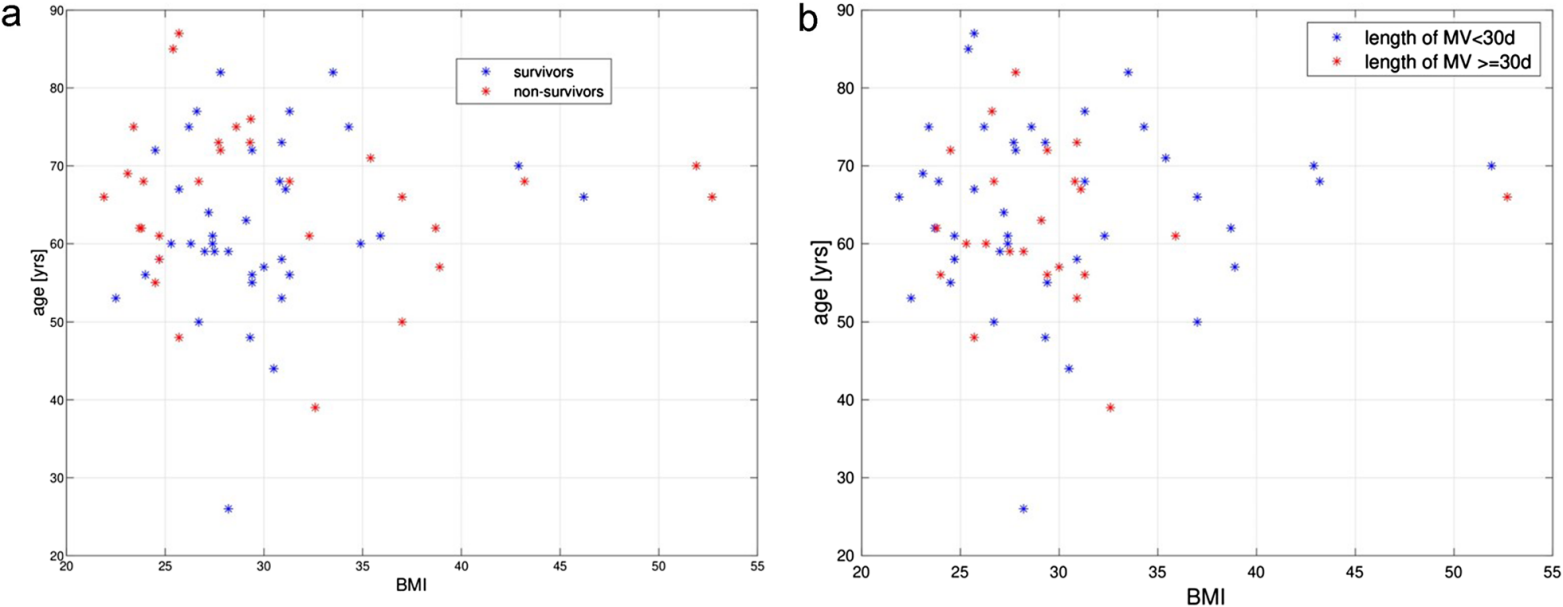
Distribution of (**a**) mortality and (**b**) length of MV across combined BMI—age data in COVID-19 ICU patient cohort

Data distribution across BMI and age revealed an apparent inhomogeneity of mortality and length of mechanical ventilation across the BMI-age plane indicating a complex, non-monotonic interaction between age, BMI and mortality, as can be seen in Fig. 1a and b. Mortality rate was 50% in the groups with a BMI below 26 and above 31. The Intermediate BMI group had a mortality rate of 16.1%. The groups divided by age exhibited a mortality rate of 34.0% for patients aged less or equal 68 years and 39.3% for those above 68 years. In our cohort, there were no significant differences between survivors and non-survivors for both BMI and age (p = 0.65 for BMI, p = 0.098 for age), indicating no significant univariate impact of each of these variables on mortality.

**Fig. 2:**
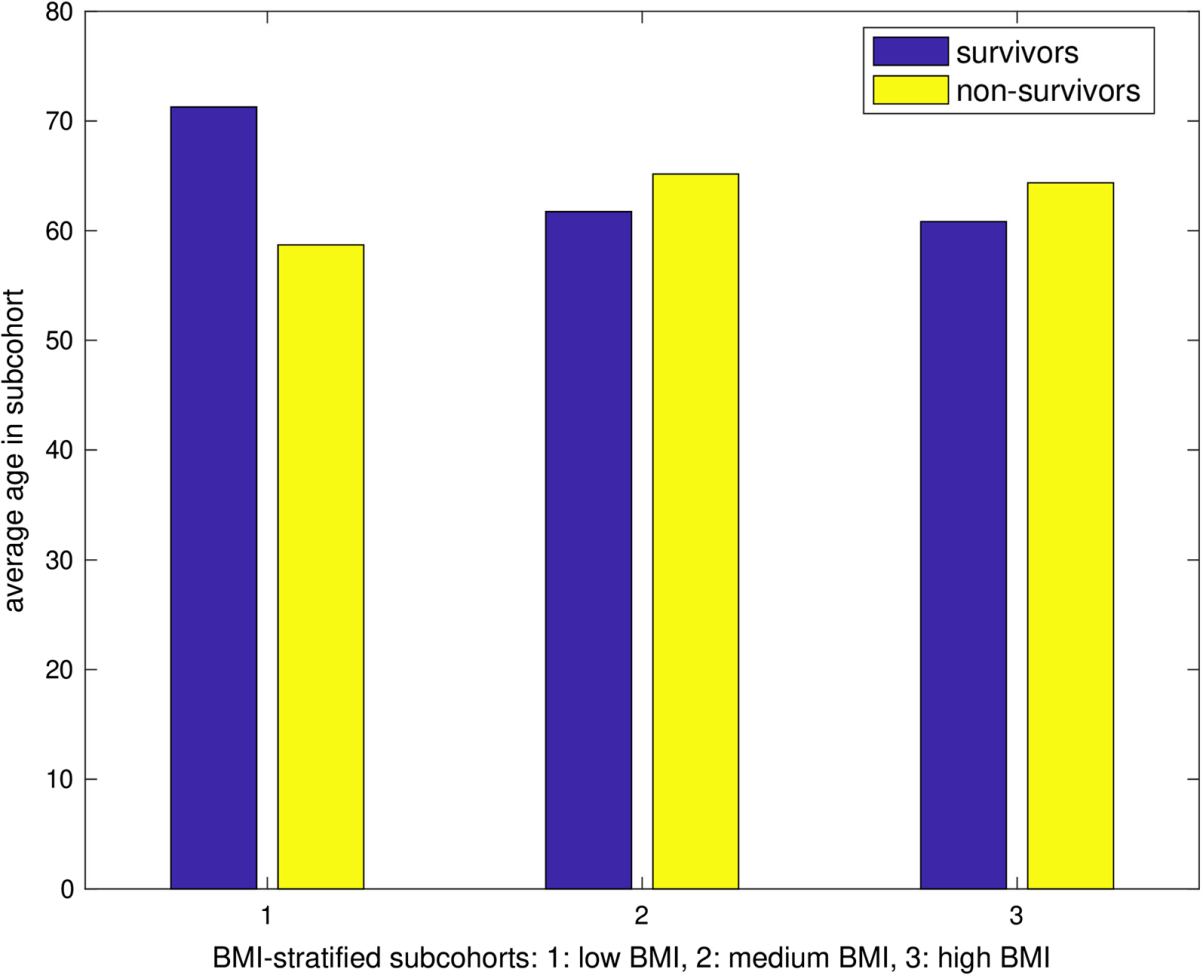
Distribution of age for survivors/non-survivors stratified by BMI

Analyzing the age distributions of survivors and non-survivors of the three BMI subgroups (low, intermediate, high) (Fig. 2) we found an apparent non-uniform impact of age on mortality. For the low BMI subcohort, mean age differed significantly (p = 0.025), whereas the differences of mean age for the medium and high BMI subcohorts were not significant (p = 0.29, p = 0.69).

To analyze the influence of BMI and age in varying combinations, we generated combined BMI–age stratified subgroups as described above, resulting in six subcohorts. Due to the variable limits of the two parameters, the sizes of the respective populations varied within each subcohort. The average numbers are given in Table 2.

**Table 2:** Mean number of patients in the six subcohorts

	BMI low	BMI intermediate	BMI high
Age low, n	14	18	15
Age high, n	7	12	9

As sizes of subcohorts depend on the splitting, they are not constant, explaining that the sum over the (univariate) means exceeds the number of patients

**Fig. 3:**
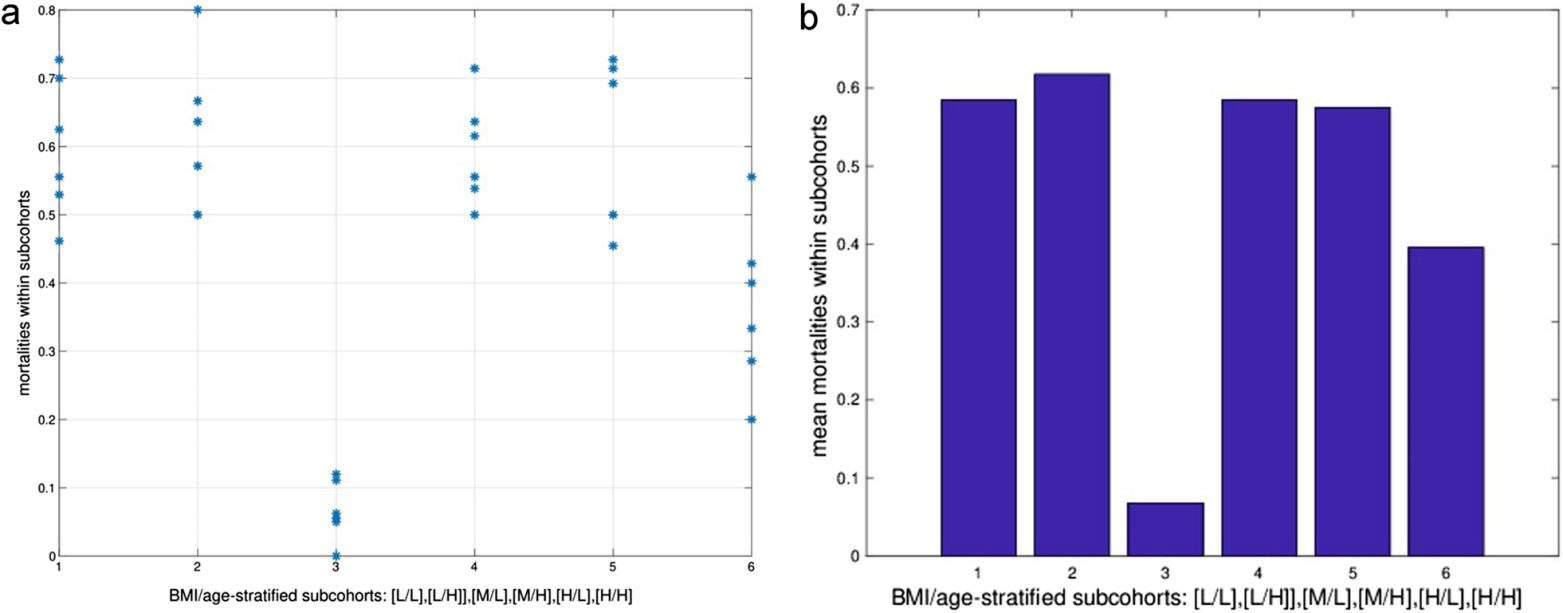
Mortality in BMI/age stratified subcohorts. **a** Mortality in subcohorts at 12 splitting levels reveals an inhomogeneous, but non-monotonic distribution of mortality. **b** Mean mortality (weighted by subcohort size) indicates outliers of a very low mortality in subcohort 3 and a slightly decreased mortality in subcohort 6. Labeling for BMI and age group: [L]: low, [M]: intermediate, [H]: high

After stratification in combined BMI/age groups, the mortality rates revealed a complex pattern (Fig. 3a and b). Whereas a group of younger patients with an intermediate BMI had a very significantly reduced risk of mortality (mean risk p < 0.001), older patients, whose BMI laid within the same range, showed a mean mortality. However, the impact of age is apparently inversed in high BMI groups: younger patients seemed to have a slightly increased risk, whereas elderly patients had a tendency towards a decreased risk (mean risk p = 0.14) compared to the overall population. Moreover, mortality in the younger patient groups was significantly (p < 0.05) increased in 4 out of 12 splitting combinations.

**Fig. 4:**
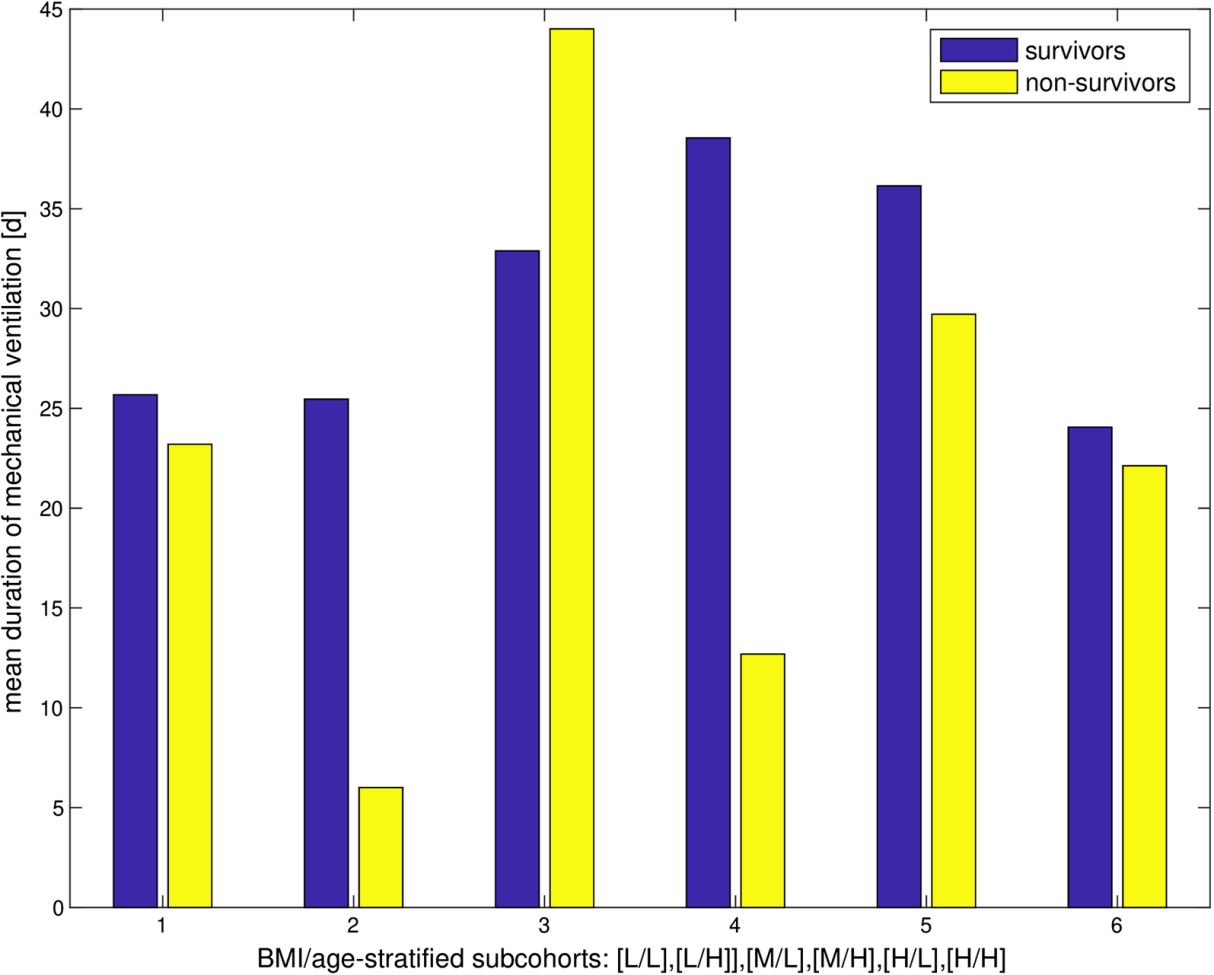
Length of MV in BMI/age stratified subcohorts. Length of MV is higher for survivors than for non-survivors in all subcohorts except cohort 3 (very low incidence of non-survival)

In contrast to our findings for mortality, the respective assessment for length of MV as secondary endpoint did not reveal significant deviations within any of the subgroups discussed above (p > 0.12). However, there is a coherent pattern for length of MV between the groups of survivors and non-survivors, as shown in Fig. 4. Except for the intermediate BMI/low age group, which stands out through its very low mortality, in all other groups the mean length of MV was overall significantly longer in survivors than in non-survivors. Here as well, the significance level between survivors and non-survivors in the intermediate BMI/low age group was not reached due to the low average number of non-survivors (N_average_ = 1.16).

### Influence of baseline values and comorbidities

To find possible explanation for the inhomogeneous mortality rates, the baseline values of diagnostic parameters were analyzed for differences between the subgroups (see Additional file 2). Analogously, we assessed enrichment of comorbidities between survivors/non-survivors for each subcohort compared to survivors/non-survivors in all MV patients (see Additional file 3). Corrected for multiple testing, both analyses showed neither significant enrichment of comorbidities nor differences in diagnostic baseline levels. Hence, we found no univariate explanation for the observed patterns in mortality.

### Differences between MV and non-MV patients

To further evaluate the observed deviations of morbidity-related patterns in other epidemiological studies and our findings in ICU patients requiring MV, we analyzed the diagnostic baseline parameters for significant differences between non-ventilated ICU patients, ventilated survivors and ventilated non-survivors.

We found that baseline values of infection parameters were enriched in MV patients compared to non-MV patients, which covers CRP (p < 0.001 for survivors; p < 0.01 for non-survivors) and PCT (p < 0.05 for survivors; p < 0.0001 for non-survivors). Baseline D-Dimers were enriched in the MV subcohort (p < 0.001 for survivors; p < 0.01 for non-survivors) compared to non-MV patients, but the p-values slightly failed the Bonferroni corrected significance level, although giving a clear trend.

**Fig. 5:**
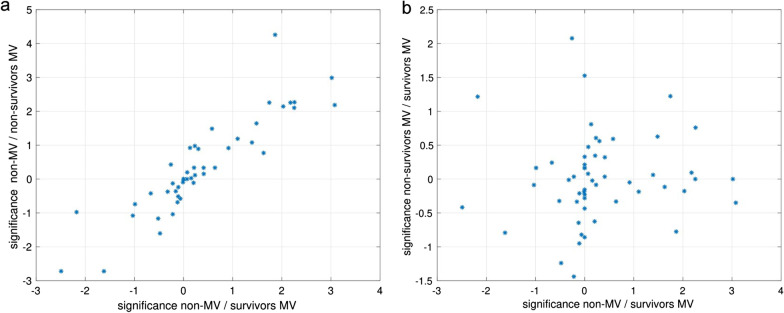
Correlation of significance levels of baseline diagnostic parameters. There is a high correlation between significance levels of baseline diagnostic parameters between (**a**) non-MV and MV patients, but no correlation when comparing (**b**) survivors and non-survivors in MV

Moreover, we found a high correlation between the log10(p)-values of non-MV vs. survivors MV and non-survivors MV across all parameters (Fig. 5a), but no correlation between non-MV/survivor-MV and survivors-MV/non-survivors-MV (Fig. 5b).

We also analyzed comorbidities for enrichment in the MV population compared to non-MV population (see Additional file 4). Apparently, sepsis was significantly enriched in MV-patient cohort compared to non-MV cohorts, independently from survival.

The distribution of enrichment of comorbidities in MV versus non-MV patients showed a high similarity to patterns received from baseline values. The log10(p)-values of enrichment of comorbidities in MV patients (independent from survival) compared to non-MV patients are highly correlated (r = 0.89), whereas the respective log10(p)-values for survivors vs. non survivors—both under MV—only show weak correlation (r = 0.42).

Considering the patients requiring MV longer than 30 days, we found significantly higher expressed D-Dimers (Bonferroni corrected p-value < 0.05) and an enrichment of ‘Diseases of the blood and blood forming organs’ in the survivors of this group.

## Discussion

The aim of this study was to get a more detailed insight into the influence of biometric covariates on the outcome of COVID-19 patients. A relevant problem within this field is the heterogeneity of study designs and strongly differing populations leading to inconsistent results. The common coincidence of several risk factors disguises a clear estimation of their individual impacts on disease evolution. A deeper computational analysis can overcome these problems analyzing the influence of a single parameter and separating it from other accompanying parameters. To show the potential of these methods we analyzed a dataset of COVID-19 patients for the influence of biometric covariates.

While other studies frequently merged patients with different disease stages also including mild courses [4–7], our study covered only patients with a highly severe health condition requiring ICU treatment. In contrast to the results reported before [11, 14–17], we found a non-monotonic impact of BMI and age on mortality and length of MV, while a univariate effect could not be determined. Furthermore, we found that typical risk factors, which are reported to be associated with increased mortality in COVID-19 patients, like elevated inflammation parameters, elevated D-dimers or sepsis [18–20], were more pronounced in patients requiring MV compared to non-MV patients. Surprisingly, the extent of these parameters could not distinguish between survivors and non-survivors in a mechanically ventilated subgroup.

These findings diverge remarkably from those, which were published before, analyzing generic COVID-19 cohorts. Especially, the differences in age-related mortality between different BMI-subgroups were a surprising observation. Many authors stress the negative effect of an increased BMI for the outcome of COVID-19 patients, partly even as a dose–response relationship [14–16]. For our population, we cannot confirm this observation. We rather found a strongly decreased risk of mortality for younger patients with an intermediate BMI. If we consider the relative mortality over all patients, this results in a U-shaped mortality risk. Interestingly, the nadir of the mortality curve seems not to lay in the "healthy" range of the BMI but in a slightly elevated range between 25 and 32, i.e. covering the preadiposity range. Holman et al. reported a quite similar distribution for COVID-19 related mortality in a cohort of English diabetes patients [21]. The causality of this finding remains unclear. Nevertheless, there are some hints that could support our results. In a large-scale study of the Global BMI Mortality Collaboration with more than 10 million analyzed participants, the risk of mortality caused by a respiratory disease, showed an U-shaped classifications with its nadir between 22.5 and 25 [22]. Thus, BMI values between 20 and 22.5 usually classified as normal weight [23], might have to be considered as disadvantageous. Underweight itself is known to be a risk factor in respiratory diseases and impairing pulmonary function [24–26].

Strangely, also the group of old patients with high BMI showed a reduced mortality, although they combine two highly endangering risk factors [11, 14–17], which are reported to be disadvantageous. Remarkably, in ARDS patients, a better outcome was found in patients with an increased BMI [27, 28]. In the end, we have to acknowledge that the sample size was small. In addition, analysis of overall 89 covariates of comorbidities or clinical/diagnostic parameters did not explain the observed mortality profiles.

These findings indicate that mortality within the ICU patient cohort is mainly driven by covariates aside from BMI and age both playing a significant role in mortality factor analysis in generic COVID-19 cohorts.

Further appropriate research considering the observed complexity regarding BMI and age needs to be carried out in larger, but more selective populations.

Although mortality is influenced by BMI and age in a non-monotonic fashion, there are no significant differences in the length of MV among the different subgroups. The only remarkable finding is a trend for longer MV durations in survivors compared with non-survivors. This pattern was also found in other observational studies [11, 29], but more pronounced in our cohort. Although we cannot fully exclude that these long MV durations were caused by severe courses of disease, it has to be taken into account that data partially include patients, who were transferred to a specialized unit for prolonged weaning, which results in longer MV durations. Non-survivors, on the other hand, die 18 days after the onset of symptoms [30] limiting MV duration.

Combining the results for mortality and MV duration, the present data could possibly indicate that the disease evolution of severely ill COVID-19 patients involves a more complex interaction of risk factors and biometric variables. This indicates that the impact factors leading to either light or severe symptoms after COVID-19 infection may be different from those responsible for the evolution to death or recovery in severe cases. These hypotheses are supported by analyses of ICU patients without MV compared to MV patients. Between these two groups, we found the risk factors for a severe course of disease, which were described before, like laboratory parameters indicating an increased inflammatory level and coagulation status [31]. Remarkably, these parameters were not able to discriminate between survivors and non-survivors as soon as MV had started. Additionally, the high correlation between all diagnostic baseline parameters and enrichment of comorbidities calculated between non-MV/MV patient cohorts as well as the lack of correlation between MV survivors/MV non-survivor cohorts indicate that the transition from non-MV to MV disease state is driven by mechanisms, which are less relevant for the following disease progression under MV.

Our study surely has limitations, which have to be considered. It has to be taken into account that the robustness of the analysis of our cohort is impaired by the small sample size, partially leading to small subgroups. To counteract this disadvantage, we used an approach for the analysis with variable group boundaries, which is based on the fuzzy logic concept. Yet, the small sample also prevented further examination on additional risk factors, like gender differences. Furthermore, the analyzed patients showed an extraordinarily high severity of the disease and complex comorbidities, which required treatment in a university hospital making it difficult to transfer the results on other populations.

Nevertheless, we claim that the risk structures of the transitions from mild to severe disease states are structurally different to the risk structures within highly severe disease states. The analysis of pooled data from all disease states, which aims to investigate risk factors for mortality, reveals the convolution of risk profiles of both disease states, dominated by the critical step, namely the step from mild to severe stage. Hence, our findings from ICU patients may not be in contradiction to the results published from large, pooled studies.

From our retrospective study, we deduce the recommendation that statistical analysis of risk factors and epidemiological/therapeutic measures should be adapted to the apparently complex and diverse disease driving mechanisms also for larger cohorts. We suggest that data analysis in COVID-19 patient cohorts should use methods that are able to find complex, non-monotonic multi-variate patterns, which are able to reflect mutual interference of parameters. This could infer the apparent complexity of the interference of disease evolution and recovery processes in critically ill COVID-19 patients.

## Conclusions

The aim of this study was to get a more detailed insight into the influence of biometric covariates on the outcome of severely ill COVID-19 patients. We found that factors for a disadvantageous outcome in this cohort were in some cases significantly different to the known ones. It seems that survival in mechanical ventilation is affected by complex interactions of covariates associated with transition from mild to severe disease stages, which are hidden in generic, non-stratified studies on risk factors. Hence, our study suggests that a detailed, multivariate pattern analysis on larger patient cohorts reflecting the specific disease stages might reveal more specific patterns of risk factors supporting individually adapted treatment strategies.

## Supplementary Information

**Additional file 1:** Table of preexisting comorbidities and diagnostic baseline value parameters. A table containing preexisting comorbidities and 54 diagnostic, baseline value parameters assessed in the ICU, i.e. the first available measurements of respective parameters, which were used to identify risk factors for a certain outcome.

**Additional file 2:** Influence of baseline values. Text showing different baseline values, which are increased/decreased within a respective subcohort.

**Additional file 3:** Influence of comorbidities. Text showing different comorbidities, which are enriched/suppressed within a respective subcohort.

**Additional file 4:** Enrichment analysis of comorbidities in non-MV patients compared to MV patients. Text showing the Enrichment analysis of comorbidities in non-MV patients compared to MV patients.

## Data Availability

The datasets of patients of University hospital RWTH Aachen, which were generated and analyzed during the current study, are not publicly available due to medical confidentiality but are available from the corresponding author on reasonable request.

## Abbreviations

ARDS: Acute respiratory distress syndrome
BMI: Body mass index
COVID-19: Corona virus disease 2019
ECMO: Extracorporeal membrane oxygenation
ICU: Intensive care unit
MV: Mechanical ventilation
PCR: Polymerase chain reaction
PEEP: Positive end expiratory pressure
SARS-CoV2: Severe acute respiratory syndrome Coronavirus 2
