# Supplementary material for: Biometric covariates and outcome in COVID-19 patients: are we looking close enough?
Source: BMC Infect Dis. 2021 Nov 4;21:1136. doi: 10.1186/s12879-021-06823-z (PMC8567725; doi:10.1186/s12879-021-06823-z)
Supplement: Supplementary file 1 — Additional file 1: Table of preexisting comorbidities and diagnostic baseline value parameters. A table containing preexisting comorbidities and 54 diagnostic, baseline value parameters assessed in the ICU, i.e. the first available measurements of respective parameters, which were used to identify risk factors for a certain outcome. [file 12879_2021_6823_MOESM1_ESM.docx]

**Additional file 1: List of diagnostic baseline value parameters and comorbidities**

To identify risk factors associated with the diverse outcome across the biometric subcohorts, we calculated differential expression for preexisting comorbidities and for 54 diagnostic, baseline value parameters assessed in the ICU, i.e. the first available measurements of respective parameters. These respective parameters and comorbidities are given in this list.

**Diagnostic baseline value parameters**

Vital signs:

- Heart rate
- Peripheral oxygen saturation (SpO2)
- Systolic arterial pressure
- Mean arterial pressure
- Diastolic arterial pressure
- Central venous pressure
- Systolic pulmonary arterial pressure
- Mean pulmonary arterial pressure
- Diastolic pulmonary arterial pressure
- Body temperature
- 24h urine output
- 24h fluid balance

Ventilatory settings:

- Respiratory rate
- Respiratory rate (spontaneous)
- Tidal volume
- Tidal volume per ideal body weight
- Tidal volume (spontaneous)
- End-inspiratory pressure (P_EI_)
- Positive end-expiratory pressure (PEEP)
- Fraction of inspired oxygen (FiO_2_)
- Inspiratory : Expiratory ratio (I:E)
- Pulmonary compliance
- Inhaled nitric oxide

Blood gas analysis parameters:

- pH arterial
- PaCO_2_
- PaO_2_
- SaO_2_
- PaO_2_/FiO_2_ ratio (P/F ratio; Horovitz)
- Base excess (arterial)
- Bicarbonate (arterial)
- Lactate (arterial)
- ScvO2

Laboratory parameters:

- Albumin
- Alanine transaminase (ALT)
- Amylase
- Aspartate transaminase (AST)
- Bilirubin
- Brain natriuretic peptide
- Creatine kinase
- Creatine kinase-MB
- Creatinine
- D-dimers
- Haematocrit
- Haemoglobin
- International normalized ratio (INR)
- Interleukin-6
- Lactate dehydrogenase
- Leukocytes
- Lipase
- Procalcitonin
- Platelets
- Partial thromboplastin time (pTT)
- Troponin
- Urea

ECMO parameters:

- Extracorporeal blood flow

**Comorbidities:**

- Certain infectious and parasitic diseases
- Codes for special purposes
- Congenital malformations, deformations and chromosomal abnormalities
- Diseases of the blood and blood-forming organs
- Diseases of the circulatory system
- Diseases of the digestive system
- Diseases of the genitourinary system
- Diseases of the musculoskeletal system and connective tissue
- Diseases of the nervous system
- Diseases of the respiratory system
- Diseases of the sense organs
- Diseases of the skin and subcutaneous tissue
- Endocrine, nutritional and metabolic diseases and certain disorders involving the immune mechanism
- External causes of morbidity and mortality
- Factors influencing health status and contact with health services
- Injury, poisoning and certain other consequences of external causes
- Mental and behavioural disorders
- Neoplasms
- Pregnancy, childbirth and the puerperium
- Symptoms, signs and abnormal clinical and laboratory findings, not elsewhere classified
- Burn trauma
- Chronic heart failure
- Chronic liver failure
- Chronic renal failure
- Diabetes mellitus
- Drug overdose
- Hematologic neoplasm
- Immunosuppression
- Inhalation injury
- Liver failure
- Pancreatitis
- Pneumonia
- Sepsis
- Transfusion-related acute lung injury
- Thoracic trauma
