# Supplementary material for: Biometric covariates and outcome in COVID-19 patients: are we looking close enough?
Source: BMC Infect Dis. 2021 Nov 4;21:1136. doi: 10.1186/s12879-021-06823-z (PMC8567725; doi:10.1186/s12879-021-06823-z)
Supplement: Supplementary file 2 — Additional file 2: Influence of baseline values. Text showing different baseline values, which are increased/decreased within a respective subcohort. [file 12879_2021_6823_MOESM2_ESM.docx]

**Additional file 2: Influence of baseline values**

We checked differential baseline values across the survivors / non-survivors in each subcohort, compared to all survivors and non-survivors. Systematic differential expressions in a subcohort compared to all MV patients are given in table 1.

Table S1: Baseline values, which are increased / decreased within the respective subcohort

| Bilirubin | Decreased (p < 0.05) | Survivors [Low BMI/high age] |
| --- | --- | --- |
| I:E | Increased (p < 0.05) | Survivors [Low BMI/high age] |
| Creatinine | Increased (p < 0.05) | Survivors [Low BMI/high age] |
| Tidal volume | Increased (p < 0.05) | Survivors [High BMI/high age] |
| SpO2 | Increased (p < 0.05) | Non-Survivors  [Intermediate BMI/low age] |

Surprisingly, the only significant difference in subcohort 3 is SpO2 for Non-Survivors despite the significant deviation in mortality.
