# Supplementary material for: Biometric covariates and outcome in COVID-19 patients: are we looking close enough?
Source: BMC Infect Dis. 2021 Nov 4;21:1136. doi: 10.1186/s12879-021-06823-z (PMC8567725; doi:10.1186/s12879-021-06823-z)
Supplement: Supplementary file 3 — Additional file 3: Influence of comorbidities. Text showing different comorbidities, which are enriched/suppressed within a respective subcohort. [file 12879_2021_6823_MOESM3_ESM.docx]

**Additional file 3: Influence of comorbidities**

**Influence of comorbidities**

In analogy to differential levels of baseline diagnostic parameters, we assessed enrichment of comorbidities between survivors and non-survivors for each subcohort compared to survivors / non-survivors in all MV patients.

Comorbidities, which are enriched / suppressed within the survivor/ non-survivor populations of the 6 subcohorts are shown in table 2:

Table S2: Comorbidities, which are enriched / suppressed within the respective subcohort

| Chronic Heart Failure | Enriched (p < 0.05) | Survivors [High BMI/low age] |
| --- | --- | --- |
| Diabetes Mellitus | Enriched (p < 0.01) | Survivors [High BMI/low age] |
| Diseases of blood and blood forming organs | Suppressed (p < 0.05) | Non-survivors [Low BMI/high age] |
| Diseases of the digestive system | Suppressed (p < 0.05) | Non-survivors  [Intermediate BMI/high age] |
| Diseases of the digestive system | Enriched (p < 0.05) | Non-survivors [High BMI/low age] |
| Sepsis | Suppressed (p < 0.05) | Non-survivors [Low BMI/high age] |

As the enrichment analysis did not result in neither significant enrichment of comorbidities nor difference in diagnostic baseline levels, corrections with respect to multiple testing show no robust significance. Hence, we cannot claim univariate explanation of the observed patterns in morbidity.
