# Supplementary material for: Biometric covariates and outcome in COVID-19 patients: are we looking close enough?
Source: BMC Infect Dis. 2021 Nov 4;21:1136. doi: 10.1186/s12879-021-06823-z (PMC8567725; doi:10.1186/s12879-021-06823-z)
Supplement: Supplementary file 4 — Additional file 4: Enrichment analysis of comorbidities in non-MV patients compared to MV patients. Text showing the Enrichment analysis of comorbidities in non-MV patients compared to MV patients. [file 12879_2021_6823_MOESM4_ESM.docx]

**Additional file 4: Enrichment analysis of comorbidities in non-MV patients compared to MV patients**

**Enrichment analysis of comorbidities in non-MV patients compared to MV patients**

Assessing the enrichment (Bonferroni-corrected p < 0.01) of comorbidities in MV-survivor / non-survivor population compared to non-MV population, we found comorbidities, which are strongly enriched in MV cohort (independent from survival) compared to non-MV patient cohort:

Table S3: Enrichment of comorbidities in MV population compared to non-MV population

|  | **Non-MV** | **MV** |
| --- | --- | --- |
| Diseases of the blood and blood-forming organs | log10(p) = -3.48 | log10(p) = -3.73 |
| Diseases of the genitourinary system | log10(p) = -2.62 | log10(p) = -3.98 |
| Diseases of the skin and subcutaneous tissue | log10(p) = -3.57 | log10(p) = -2.22 |
| Endocrine, nutritional and metabolic diseases | log10(p) = -3.83 | log10(p) = -3.3 |
| Symptoms, signs and abnormal clinical and laboratory finding | log10(p) = -3.92 | log10(p) = -3.98 |
| Sepsis | log10(p) = -4.8 | log10(p) = -6.3 |

Apparently, Sepsis is a syndrome, which is significantly enriched in MV-patient cohort compared to non-MV cohorts, both for survivors and non-survivors.
